# Health Promotion in Debate: The Role of Women Leaders in the Favelas of Rio de Janeiro, Brazil

**DOI:** 10.3390/ijerph20115926

**Published:** 2023-05-23

**Authors:** Nilza Rogéria de Andrade Nunes, Dais Rocha, Andréa Rodriguez

**Affiliations:** 1 Social Work Department, Catholic University of Rio de Janeiro, Rio de Janeiro 22451-900, Brazil; 2 Collective Health Department, University of Brasilia, Brasilia 70910-900, Brazil; dais.rocha@unb.br; 3 School of Dentistry, University of Dundee, Dundee DD1 4HN, UK; a.rodriguez@dundee.ac.uk

**Keywords:** women leaders, favela, health promotion, participatory methodology

## Abstract

This study aimed to discuss the actions of women leaders in favelas in Rio de Janeiro, Brazil, regarding the health promotion of people residing in territories affected by urban violence and inequalities. The understanding of social determinants of health (SDH) is not unequivocal and challenges us to expand our practices in health promotion and equity. A mixed-methods study was conducted with 200 women living in 169 favelas of Rio de Janeiro between 2018 and 2022. Questionnaires and semi-structured face-to-face interviews followed by thematic analysis were conducted. The analysis focused on the socio-demographic profile, community activism, and health promotion strategies undertaken by these groups, expanding knowledge on the experiences of these leaders in confronting social injustices. Results showed that participants performed health promotion actions in their communities by 1. strengthening popular participation and human rights, 2. creating environments favorable to health, and 3. developing personal skills towards social participation in policy design through mobilizing health services and third-sector organizations. With the limited presence of government public agents in these spaces, participants assumed roles as managers of local demands, and, by means of resistance, intersectionality, and solidarity, they transformed this micro-power into the potential for social transformation.

## 1. Introduction

Examining the favelas in Rio de Janeiro, Brazil, from the perspective of public health prompts a debate on the social determinants of health and their intersection with the efforts of individuals engaged in processes of mobilization and resistance [1]. It is known that the social inequalities evident in the lives of a large part of the Brazilian population have a great impact on the country’s social, economic, and technological development. The COVID-19 pandemic has exacerbated poverty in Brazil [2]. As a result, considerably more people have been excluded from basic human rights, such as education, work, housing, security, and especially, health, with some groups being relegated to invisibility. People living in the favelas of Rio de Janeiro have become more prone to developing health problems due to life circumstances and poverty and have more difficulty accessing health services due to the barriers imposed by drug trafficking gangs that control these territories and limit the circulation of residents from one area to another. However, the leadership of women in these territories of inequalities has gained recognition and prominence in academic studies [3,4].

When referring to the work of women leaders in the favelas of Rio de Janeiro, we are acknowledging the construction of politically engaged individuals that is shaped every day. These women leaders are activists who are emblematic of the daily struggle of a segment of our population living under a development model that imposes limitations on their access to rights and perpetuates inequalities among the most impoverished individuals. Through the transformation of their daily lives, these women embody a story of relentless advocacy for human rights and efforts to raise awareness about inequalities, thus reinventing political engagement [3,4,5]. These women carry within themselves the restlessness of a silent revolution in behavior [6], whose freedom is announced every day as they routinely break with attempts to silence them, presenting their narratives and experiences that echo their voices.

In order to understand what “favela” means, we will adopt the conceptual framework created by Valladares [7], which has been embraced by several authors and shared by several authors [8,9]. This concept establishes a dialogue between the irregularity of urban space occupation by the poor, and the presence of urban violence intertwined with cultural expressions that shape the identity of the urban territory of the underprivileged—favelas as a “city within the city”, enclaves, and partitions symbolizing socio-spatial segregation. However, there is also a representation of favelas that encompasses a sense of community, where a multifaceted and plural reality becomes evident.

The favela plays an essential role in the city of Rio de Janeiro, encompassing geographic, economic, social, and political dimensions. It is part of the city, although historically its existence has been denied due to its negative image, associated with insecurity and violence. This homogenizing vision and a discourse focused almost exclusively on absences result in an image of a favela where residents do not recognize themselves as active and performing agents, inserted in the time and space of the city—hence, as citizens with rights. Characterized by physical and symbolic boundaries, the favela constitutes areas of separation and interaction, where social-spatial practices that are manifested in the landscape define places and forms of belonging and express territorialities and forms of urban appropriation.

The concept of health promotion starts from an expanded conception of the health-disease process and its determinants, and proposes the combination of technical and popular knowledge, advocating for the integration of technical and popular knowledge, as well as mobilizing institutional and community resources—both public and private—to address and resolve health-related challenges [10]. Therefore, health promotion seeks to overcome the biomedical model by employing comprehensive approaches that encompass political dimensions, personal and collective skills development, community engagement, environmental considerations, and the reorientation of healthcare services [11].

Exploring the relationship between health promotion and the experiences of these leaders in confronting social injustices in these territories allowed us to broaden our knowledge about the SDH in their commercial, social, cultural, and political dimensions. Health inequity has been addressed through various studies and conceptions. According to Buss and Filho [10], one analysis that is considered relevant emphasizes the “physical-material aspects” in the production of health and disease, understanding that income differences influence health due to individuals’ lack of resources and the absence of investments in community infrastructure (education, transportation, sanitation, housing, health services, etc.), resulting from economic processes and political decisions. A second analysis is based on the psycho-social factors that affect individuals and groups based on the unequal experiences that cause stress and damage to health, and a third is based on living conditions and the extent to which links and associations between individuals and groups are established.

These different approaches demonstrate that the understanding of SDH is not unequivocal. The International Commission on Social Determinants of Health (ICSDH), established by the World Health Organization (WHO) [12], has proposed that the SDH should include the experiences of the individual in relation to education, economic situation, employment and work, housing, and environment, as well as efficient systems for the prevention and treatment of diseases. This proposal remains the most widely disseminated worldwide, based on the Rio Political Declaration on Social Determinants of Health [13]. The ICSDH recommended that interventions to address these determinants are essential for societies to be inclusive, equitable, economically productive, and healthy. But the debate continues on the nomenclature, frameworks, metrics, and pathways of how the SDH act in the health-disease-care process [14,15,16].

Discussions revolving around determinants and social determinants of health have explicitly recognized the contributions from other critical perspectives on health, particularly from countries in the Global South [14,17]. Advocates of the social determination approach argue that supplanting the ill-health model requires changing the modes of production and consumption in our society. Currently, this view is being taken up again using propositions from the commercial approach to the social determinants of health [18,19,20]. Currently, this viewpoint is being revisited through propositions originating from the commercial approach to social determinants of health [18,19,20]. Additionally, as a means of fostering political will for change, proponents of this approach advocate shifting the focus away from governmental and multilateral actors and acknowledging the central role of civil society movements and/or organizations.

Finally, in this study, we argue that one of the meanings that the SDH can take on is to steer the implementation of public policies from the perspective of promoting equity in health and reaffirming health as a right, unveiling conflicts of interest and disputes in order to safeguard it.

The objective of this article is to understand the role and strategies of women leaders in the favelas of Rio de Janeiro, Brazil, in addressing health inequalities among people living in territories affected by inequalities.

Given the scenario context in which social and health inequalities are increasingly expressed among the most vulnerable populations, identifying and recognizing ways of living and operating in this context is essential if we want to effectively contribute to the reduction of inequities. It is crucial to identify and recognize ways of living and operating within this context to effectively contribute to reducing inequities. Gathering information and contributing to the visibility of who they are and the practices of women who are identified and publicly recognized as community leaders is fundamental. Women who are identified and publicly acknowledged as community leaders are essential. There is much to be learned about their actions in popular spaces, their work with public services, their advocacy in the system of guaranteeing rights, and in the promotion of health.

## 2. Materials and Methods

This study adopted a community-based participatory research (CBPR) approach. Adhering to the principles of CBPR, which prioritize community empowerment and broad participation, an advisory group comprising female community leaders was formed to contribute throughout all stages of the research, including the design phase [21]. CBPR embraces collective efforts to achieve strong participation among people from the communities and uses research processes to build on the strengths and priorities of the community as a way of developing strategies to improve health and social equity [21]. CBPR emphasizes collective efforts to foster active community involvement and utilizes research processes to build upon community strengths and priorities, ultimately developing strategies to improve health and social equity [21]. By adopting these principles, CBPR has established an important approach to question the power relationships in research processes through an orientation that is community-based and community-directed rather than just community-placed [21]. Researchers adopting these principles have contributed to increasing health equity by integrating non-academics into engaged research processes. An initial group of twenty-five participants was contacted by the principal investigator, who had conducted previous research with them. The other participants were identified using snowball sampling [22]. In the snowball method, the initial invited participants nominate new participants, and so on [23]. This working process can be described as obtaining data or information about characteristics, actions, or opinions from a certain group of people, indicated as representative of a target population, by means of a survey instrument. This is a non-probability sampling technique that uses reference chains in a kind of network [24]. The project was submitted and approved by the Ethics Committee of PUC-Rio University (No. 44/2018) and was supported by the National Council for Scientific and Technological Development (CNPq) and Fundação Carlos Chagas Filho de Amparo à Pesquisa do Estado do Rio de Janeiro (FAPERJ), number 268327.

The active search for female leaders in the favelas was based on contacts, approaches, referrals, and the principal researcher’s presence in places frequented by these women such as civil society forums and community networks. Therefore, the identification of potential participants was based on the involvement of these women as active participants in their communities, perceived and publicly recognized as leaders by community-based organizations, and people living in their communities for at least one year. This recognition and their socio-political action were our main criteria for participating in this study. The study did not have a specific location, as the search led us to women from various regions of Rio de Janeiro. As such, there were no physical boundaries within the city that impacted the identification of participants.

The study took place from 2018 to 2022 and involved 200 women living in 169 favelas in Rio de Janeiro. Due to the COVID-19 pandemic and the resulting lockdown measures that occurred during the research, face-to-face interviews were conducted with 124 women, while the remaining were interviewed through digital platforms such as Zoom, Google Meet, or video calls via WhatsApp, with appropriate modifications to the informed consent form.

The instrument used for data collection was a semi-structured interview with the use of an electronic questionnaire (on the Google Forms platform) composed of 35 closed multiple-choice questions and 8 open questions, developed by the research team. The 35 questions were divided into 7 blocks of questions: (1) sociodemographic profile; (2) community activism; (3) health promotion strategies (categories extracted from the mapping of the Brazilian National Health Promotion Policy, tested nationally across the five regions of the country through a study by the Ministry of Health); (4) spaces for participation; (5) evaluation of the work carried out; (6) power and empowerment; (7) feminism. For this paper, we focused on the results and analysis of the first, second, and third blocks. The data were recorded in a database in the Statistical Package for the Social Sciences (SPSS), version 2017. The questionnaire was designed by the lead researcher with the participation of an advisory group from the community and was piloted to allow for necessary changes. It covered the key themes: socio-demographic profile; community activism; and health promotion strategies.

Once the interviews and questionnaires were concluded, the data was analyzed using the charts generated from the closed-ended questions, while the open-ended responses were examined, related, and grouped into macro-categories to facilitate data interpretation and dissemination [25,26]. This process followed the six stages of analysis as proposed by Braun and Clarke [26]: (1) familiarization with the data; (2) generating initial codes; (3) looking for themes; (4) reviewing the themes; (5) defining and naming the themes; and (6) producing the report [25].

## 3. Results

### 3.1. Socio-Demographic Profile

Table 1 shows participants were between 17 and 65 years old; most were black (91%); mothers (66%); married (42%); and they were the primary income earners in the family (58%). Regarding religion, 31% declared themselves evangelical, having no religion (18%), Catholic (18%), and others (33%). Sixty-two percent had access to higher education. They work in education, health, social assistance, the environment, and others. All the interviewees are part of collective spaces for political and social participation (100%). In their struggle and social resistance, they continue their activities, assisting families and welcoming those who need support and sensitive listening.

Brazil was structured by racism, and the slave system lasted for over 300 years. “It is a historical process that over time has determined people’s social places according to race or ethnicity” [27]. In this way, the favelas and peripheries have been places that impose themselves on Black women. And this is reflected in this study, since in the self-declaration of race/color, they correspond to 92% of the interviewees. This data reflects the historical gender inequalities through which social indicators confirm. Women are affected by factors such as occupational segregation in the labor market, educational opportunities, and lower salaries in similar occupations. The employment level of women is substantially lower than that of men. While men’s employment level was 61.4%, women’s was 41.2% in 2020. But when it comes to Black women, this picture worsens. According to the Synthesis of Social Indicators by the IBGE [27], 63% of the households headed by Black women are below the poverty line, while Black women still receive less than half the salary of White men in Brazil.

Regarding education, 62% of the 200 study participants had access to higher education, broadening their educational qualifications. This means that 29% have completed a university degree and 15% have a post-graduate degree. This data is significant as it reflects the increasing participation of Black men and women from favelas in universities since the 1990s, made possible through access and retention policies, including racial quotas. The experience of attending a university has enhanced their ability to navigate the city, granting them more autonomy and freedom to build the paths that lead them to improve their own lives, those of their families, and their communities. Nevertheless, while they study or have studied, they have not abandoned their place of belonging—the community, here in the genuine sense that the term suggests: the place of their identity, of neighborhood relations, and of solidarity. On the symbolic level, the relationships that certain social groups develop with their territories of living and housing, in the case of female leaders in the favela, are configured as a space of affective investment and where individual and collective identities are formed [27,28]. The research participants are actively engaged in their educational trajectory and have brought knowledge to their places of action and residence.

The division between housework and childcare is reflected in this group of women. When asked about their motherhood, 66% said they were mothers, and concerning their relationship status, 42% of our female participants said they were married. The living conditions of the working-class residents of the favelas and surrounding regions are reflected in the study, where unemployment and underemployment rates are more pronounced. However, it is by seeking ways to improve their lives that they sustain their homes and their families.

In relation to the literature on the sexual division of labor, it is important to consider multiple variables. The sexual division of domestic labor and disadvantages in the world of work are complementary facets of the inequalities between women and men today [29]. Gender inequalities have shown that the practices and values that underpin a sexual division of labor founded on conventional notions of femininity and masculinity impact women’s participation in all spheres of society [30]. However, although Black women are the most impacted by extreme poverty, poverty, and vulnerability, our study shows that these activists do not resign themselves to this condition. Their voices and actions resonate. In the case of these women, they play a crucial role in assigning responsibilities in both private life and the public sphere [30].

Religious life occupies a strategic place in popular spaces. Where public policies are poorly provided, other organizations come into play and, beyond the formative dimension of religion, they provide a sense of belonging and, furthermore, reveal the role of faith in the lives of these women from the favelas. This is reflected in community life, where historically the Catholic church was prevalent through the grassroots ecclesial communities, but nowadays it is the Evangelical/neo-Pentecostal churches that take center stage. Of our interviewees, 31% are Evangelical, followed by 23% with no religion, 14.6% Catholic, and the remaining 31.4% are distributed among Umbanda, Candomblé, and Buddhist religions, among others.

The data regarding religion was surprising, considering that previous studies have shown that the Catholic Church was a determining factor in the formation of leaders in previous decades through pastoral work and its own activities in the favelas and peripheries. In this sense, the intersection of religion and education reveals that the leaders with lower levels of education (particularly the older ones) are those who declared themselves to be Catholic. However, among participants who declared having no religion, the distribution varied across different levels of schooling. Religions of African origin, on the other hand, were more apparent among those who had completed higher education and post-graduate studies. 

### 3.2. Community Activism and Health Promotion

Table 2 shows the areas of action where participants placed their community activism for health promotion. Categories from the Brazilian National Health Promotion Policy were used, and participants indicated the options they felt were most closely associated with their work. The following three areas of action were the most frequently mentioned by participants: violence prevention and encouragement of a culture of peace in the communities; promotion of sustainable development; and physical activities and body practices.

The presence of women in community activism is not new, but its growth is an important element in the daily life of the favelas, whether because of women’s protagonism, the increasingly visible struggle against racism, or access to education, among other motivations. If the initial motives were the fight for improvements in the living conditions of residents in favelas, such as the construction of day-care centers, paving, and sanitation, among others, they are now brimming with demands that they embrace as their own. Notably, these changes and improvements are increasingly led by women who promote an “aquilombar” (from quilombola/quilombo) [31], which can be understood as a sense of dreaming, fighting, and hoping for social justice. In this sense, one of our participants said:

*People feel valued when they are aware that their history and memory are of great importance for the caring of community, for the common good that belongs to everyone. This increases people’s confidence and autonomy to make changes with the certainty that all this leads to a healthy environment*.

(research participant)

The idea of quilombo today takes on the meaning of an ideological instrument against forms of oppression, shifting from a clandestine institution to a symbol of resistance [32], as in the context of the favelas, for example. According to Beatriz Nascimento, quilombo “is not an idea located in the past but a cultural continuum of agglutination, in the sense of aggregation, community and resistance for the recognition of humanity and preservation of the cultural symbols of Black people” [31].

The leaders do not work alone—100% of our participants were working in collaboration with different stakeholders and taking part in collective spaces:

*We must work together towards the development of individual and collective skills for all of us, for community strengthening, with women as protagonists in this process*.

(research participant)

As such, networking (whether created within the favela or outside of it) and participation in social movements and in the struggle for public policies that address the needs of the favela and its residents both individually and collectively. These efforts serve as a powerful reminder of the significant contributions made by these women and the necessity of recognizing and acknowledging their endeavors.

This observation highlights another key element of their practice, as they were not only working to benefit the lives of those living in favelas but also encompassed broader urban contexts. In this sense, we are interested in learning about the participation of social activists who live in the favelas in this micropolitical action of mobilization and negotiation with public authorities. These women, subjects with agency, are creating certain repertoires to confront the unequal structure to which they are permanently subjected. They recognize that the state does not act to ease the conflicts posed by inequalities or to guarantee better ways of living in the favelas, but they are not complacent about it.

Participants stated that they were working to promote health; 92.5% recognized that their practices were contributing to the promotion of health in their territories through community activism. These efforts are related to the improvement of the SDH, which links health issues with collective struggles for civic engagement and human rights:

*Because by contributing to the promotion, information, and access to fundamental human rights, such as: citizenship, education, assistance, participation, leisure, etc., the work I do ends up promoting health, from the perspective of an integral and multifactorial health*.

(research participant)

The statements that corroborate their claims were diverse, including guidance and referral to health services as well as the provision of health-related sessions to increase awareness of wider health issues.

Following the guidelines of the Brazilian National Health Promotion Policy (PNPS) [33], our participants were asked to point out aspects in which they identified as having a role, as well as which strategies they used in their daily actions.

One of the core pillars of the PNPS is to promote a culture of peace and prevent violence. This was highlighted because the lens of the instrument and the analysis of the actions of these women from the favelas were according to the PNPS. In this sense, 71.1% of the study participants declared they act on violence prevention and stimulate a culture of peace in their communities. This is extremely important as the urban violence generated by the presence of drug trafficking gangs in these territories affects different aspects of the health of the residents, such as mental health issues and limited access to health services.

*The Government’s actions, the State, the military police kill the youngest and sickens the whole family*.

(research participant)

It was found that 46.8% of participants reported that their actions were associated with the promotion of sustainable development, 40.3% in physical activities and body practices, 36.3% in the health care network, and 35.3% in healthy eating, among other fronts of action on a smaller scale.

### 3.3. Health Promotion Strategies

Table 3 shows that participants used several strategies to achieve health promotion in their territories, such as strengthening popular participation in political spaces of decision making for policy design, mobilization of residents towards the defense of their rights, forming partnerships and participating in intersectoral actions, and developing their personal skills, among others.

The participant’s statements below are important as it helps us understand how the practices and attitudes of female leaders favor ways of life in groups and communities where participation in the co-design of public policies is precarious and insufficient, considering the priority themes of the PNPS and some of its objectives.

*We live in a place where you don’t have access to culture and citizenship. Bringing people’s participation encourages people to seek for their rights*.

(research participant)

*I am a local health counselor. I help publicize and raise awareness of rights and the importance of people’s participation in the fight for them*.

(research participant)

As facilitators and mediators of a permanent dialogue with public authorities, and agents of resources, these participants skillfully develop an infinite number of strategies capable of favoring better ways of living for their communities. Their humanized approach to action and change is something that should be highlighted:

*I encourage healthcare in all aspects, with the body, health care for others. Because I believe in eye-to-eye policy, affection and listening*.

(research participant)

*In addition to facilitating the links between residents and the public health system, I also guide them and encourage self-care within the concept of general well-being as being as an extended health*.

(research participant)

Intersectionality, interdisciplinarity, and social participation are key principles embedded in participants’ practices and paths. The action of community leaders at local levels is aligned with the reality and challenges they face in their lives and work [34]:

*We live in a place where you don’t have access to culture and citizenship. Bringing people’s participation encourages people to seek rights and make change*.

(research participant)

## 4. Discussion

Brazil was structured by racism, and the slave system lasted for over 300 years. “It is a historical process that over time has determined people’s social places according to race or ethnicity” [35]. In this way, the favelas and peripheries have been inhabited mostly by Black and poor people. And this is reflected in this study, since in the self-declaration of race/color, 91% of the interviewees reported they were Black/brown. This data reflects the historical gender inequalities through which social indicators confirm. Women are affected by factors such as occupational segregation in the labor market, educational opportunities, and lower salaries in similar occupations. The employment level of women is substantially lower than that of men. While men’s employment level was 61.4%, women’s was 41.2% in 2020. But when it comes to Black women, this picture worsens. According to the Synthesis of Social Indicators by the IBGE [36], 63% of the households headed by Black women are below the poverty line, while Black women still receive less than half the salary of White men in Brazil.

Being a Black woman and a favela resident in Rio de Janeiro leads to triple discrimination since the stereotypes generated by sexism, racism, and gender inequality put them on the edge of subordination. Therefore, we see a confluence of oppressions that fall upon these and other women who live in the favelas. Accordingly, we include the dimensions of gender, race, and class that bring us back to the notion of intersectionality proposed by Crenshaw [37]. From the perspective of the favelas and peripheries, Grada Kilomba supports bell hooks’ understanding that “to be on the margin is to be part of the whole, but outside the main body” [38]. She also adds that “the margin should not only be seen as a peripheral space, a space of loss and deprivation, but rather as a space of resistance and possibility” [38]. Consequently, where there is a “situation of scarcity of resources in the most diverse forms, there is also a place of potency” [31].

The favela is a locus where multiple forms of violence and rights violations are committed against its inhabitants, and it becomes a prime space for health promotion. The data in Table 1, which showed that almost all participants were Black and residents of favelas, suggest a correlation with their choices of areas of action (Table 2) that shows a higher concentration of work on the prevention of violence and the strengthening of a culture of peace. Considering that the favelas in Rio de Janeiro are popular spaces marked by urban violence, notably by the armed control of drug trafficking gangs and the frequent violent incursions by the public security policy of the so-called “war on drugs”, this context has shaped the role of these women fighting for a culture of peace. It is well known how the military police have a different attitude when interacting with Black people and/or entering favelas in comparison with other areas of the city. In these territories, there are different codes of conduct by the security forces, and a series of rights violations, such as abuse of authority and power, violent body searches of residents, including children (and with no clear reason), and the summary execution of young Black people who did not resist arrest, among others.

Under these circumstances, the women from the favelas who have become key figures within their communities have identified the urgent demands of the community regarding the enhancement of their chances of existing by ensuring all their rights, including health, and have therefore developed strategies and actions in health promotion (Table 3), namely: the creation of environments favorable to health; the strengthening of community actions; and the development of personal skills towards social participation in policy design. These actions are compatible with some of the PNPS guidelines: encouraging intra- and intersectoral cooperation and incorporating health promotion interventions in the health care model by means of intersectoral actions.

The second-largest area in Table 2 chosen by the participants to carry out their work was the promotion of sustainable development, which is another area that is related to the reality of those living in favelas in Brazil, as these territories were historically neglected and usually receive less government investment in education and health equipment and resources for their population. Another element of correlation presented in Table 3 regarding the strategies adopted by the female leaders towards health promotion and health equity is the strengthening of popular participation and community mobilization for the defense of human rights. These responses seemed to be well linked with the race aspect (Table 1) and the areas of action (Table 2) related to the prevention of violence and the strengthening of a culture of peace. The strengthening of popular participation towards a clearer understanding of human rights and utilizing formal ways to claim their rights is an important strategy to change the mentality of citizens of the second category, a Brazilian expression that is used to diminish and discriminate against Black and poor people living in favelas due to their locality of residence and skin color.

Looking closely at women’s practices through the lens of health promotion has provided us with important lessons. The understanding that they bring to their actions involves an important interpretation of health as a right that encompasses many dimensions and is not limited to the absence of disease. On the contrary, upon receiving an answer from our participants, almost in their entirety, that they recognize the contribution of their actions in the community as health promoters, they also demonstrate the self-knowledge that their work has an impact and promotes change in people’s lives. According to Bandini and Germani [39], health promoters are considered to be people, organizations, and groups from various sectors who work to promote health, regardless of their professional designation. Accordingly, they bring knowledge, skills, and proposals for the promotion of objective and subjective conditions in their ways of doing health in accordance with the principles and values of the PNPS.

The mobilization and fostering of community participation and actions that value local culture and social resistance movements accompany the work of the participants, rewarding their collective achievements and actions. Mediation in the access to services in addition to advocacy in defense of their local agendas is also reflected in their Health promotion practices, as defined by them: people feel valued when they are aware that their history and memory have a great relevance to care [...]; it increases people’s confidence and autonomy, and certainly, all this contributes to a healthy environment in which people realize the value they possess [...]; the work carried out is to provide information to young people about good nutrition, healthy living, and the right to health; one of the things that is taught is basic sanitation, waste management, and organic products; a professionally trained person will be able to access better opportunities; among many other answers.

The actions of favela women, as members of civil society, are critical to the promotion of health and equity in these territories. The community activism of participants seemed to be reinforced by their search for the guarantee of social rights for all favela residents, alongside their own experiences as Black women and residents in these territories as well. Recognizing the systematic and historical violation of rights to which people from favelas are subjected because of where they live and their skin color seems to be a strong motivation that drove these women to carry out work on health equity and social justice. This study corroborates the central role of civil society organizations as a determinant of the health-illness-care process.

## 5. Conclusions

The most effective ways to promote health and decrease health inequities can be achieved by creating more just economic, social, and political conditions for those living in territories marked by extreme inequalities such as favelas in Rio de Janeiro. Practical strategies are desperately needed to reduce poverty, racism, and powerlessness.

The work of leaders in popular spaces marked by violence and human rights violations requires skill, creativity, courage, daring, and resistance. Their struggles translate into the recognition of themselves and favela residents as subjects with rights, the reaffirmation of the values of justice and social participation that guide the humanization of bodies, and the construction of strategies to overcome economic and social inequalities.

The activism of these women has been consolidating over the decades, year after year. Building their own modus operandi to perform their work following a bottom-up approach, they oppose the classic and hegemonic vision of women restricted to the private space of home and care. They act locally and globally based on an idea of a city that is for everyone, regardless of social class, place of residence, or skin color. When it is evident that there is a process of feminization of power underway in popular spaces, and with it a diversity of experiences led by women that have a direct impact on the ways of living in the favelas, we can recognize the importance of this leadership exercised by women in the implementation of a political practice.

Intersectionality runs through the work of the participants and is an expression of the fact that gender, race, and class, associated with their geographical and environmental context, are translated in this group as existence and resistance to unequal power structures. The lived experience and the different ways of confronting the reality that involves their personal lives and that of other residents in their communities show the struggle of these women in a sphere that is public, but not state.

The systemic violence to which Black women have historically been subjected will no longer silence them. They routinely break the veiled silence of colonial patriarchy, becoming subjects of this process of transition from silence to the revolutionary act of speech, in the words of bell hooks [40]. According to this author, women may develop negotiated power since multiple spaces of participation and coordination with the public authorities are produced, an initiative promoted by local stakeholders. Historically immersed in perverse power relations, the women we refer to here are treading along possible paths to a social construction based on recognition, solidarity, appreciation of the other, and understanding of a society that lacks understanding of the social meanings that reflect their past and present history.

Increasingly educated, in addition to their work, they become a reference for many others. Driven by networking, they coordinate with other groups—forums, collectives, and councils—which strengthens them in the construction of their narratives and practices, besides amplifying the visibility of their actions.

Therefore, the fact of recognizing that health promotion practices produced by community members are capable of activating resistance, reducing individual accountability and guilt, while “bringing about changes in a future perspective that aims at a more just, cooperative, solidarity-based and sustainable society, seems to us, to be the most relevant, possible and desirable to which we can aspire” [41]. Balanced health requires safe and secure conditions, such as housing, income, public transportation, proper environmental conditions, and job security, which are just some of the elements necessary for a healthy life [11,41].

Regardless of the differences in terms of action where their practices are consolidated, these women seek to respect each other, accepting and/or valuing knowledge and choices. They are subjects, with consciousness “of themselves” and their place inside a collective, acting towards equal solidarity and commitment to new paths of conducting politics with and for people. These women are occupying spaces of social participation, whether in civil society movements, through councils of rights, or even in their grassroots organizations.

Mindful of the motto “we for us” and solidarity in the favela, these women promote health by understanding the social determinations allied to broader structural issues that have a direct impact on the absence of public policies to ensure the basic rights of favela residents. The presence of the government in several favelas in Rio de Janeiro has been especially marked by the existence of security policies. As a result, we are witnessing the genocide of young Black men committed by the state, whose practices are fed by classism, racism, and urban segregation. The community activists represented here do not remain silent; in a movement of struggle and social resistance, they continue their activities, providing assistance to families and welcoming those who need support and sensitive listening. In this way, their struggles translate into the recognition of civic engagement, the reaffirmation of the values that guide the humanization of bodies, and the construction of strategies to surmount inequalities.

## Figures and Tables

**Table 1:** Sociodemographic profile. Proportional distribution of research participants according to the sociodemographic characteristics based on the sample number (n = 200).

Variables	f	%
Age Group		
Up 17 years old	1	1%
Between 18 and 24 years old	19	10%
Between 25 and 34 years old	38	19%
Between 35 and 44 years old	39	20%
Between 45 and 54 years old	38	19%
Between 55 and 64 years old	37	19%
65 years or more	17	9%
Not informed	12	6%
Race		
White	16	8%
Black/brown	182	91%
Yellow/Indigenous	2	1%
Level of Education		
Completed elementary school	3	2%
Incomplete elementary school	4	2%
Completed secondary education	48	24%
Incomplete secondary school	11	6%
University degree	58	29%
Incomplete university degree	47	24%
Postgraduation or more	29	15%
Religion		
Evangelical	75	37%
Without religion	48	24%
Catholic	29	14%
Candomblé	13	6%
Espiritism	13	6%
Umbanda	10	5%
Buddhism	2	1%
Relationship		
Married	83	42%
Divorced	1	1%
Dating	22	11%
Single	85	43%
Widow	9	5%
Number of children		
Without children	67	34%
1 child	46	23%
2 children	46	23%
3 children	26	13%
4 children or mores	15	8%

**Table 2:** Areas of action from research participants to promote health.

	%
Violence prevention and encouragement of a culture of peace in the communities	71.1
Promotion of sustainable development	46.8
Physical activities and body practices	40.3
Reorientation of care in the health sector towards an humanized approach	36.3
Healthy eating	35.3
Work life balance	30.8
Reduction in the abuse of alcohol and other drugs	29.9
Smoking prevention	15.9
Reduction in morbidity and mortality due to accidents	5

**Table 3:** Strategies adopted by participants.

	%
Strengthening of popular participation	72
Mobilization and defence of rights	43
Partnerships/collaborations/intersectoral action	62
Development of personal skills	46
Continual dialogue with health services	46
Improved access to resources/services	45
Creating healthy environments	42
Leadership support and training	40
Communications and social media	38

## Data Availability

Research data are unavailable due to privacy or ethical restrictions.

## References

[1] Porto M.F.d.S., Cunha M.B.d., Pivetta F., Zancan L., Freitas J.D.d. (2015). Saúde e Ambiente na Favela: Reflexões Para Uma Promoção Emancipatória da Saúde. Serv. Soc. Soc..

[2] Neri M.C. (2022). Mapa da Nova Pobreza.

[3] Nunes N.R. (2018). Mulher de Favela: O Poder Feminino em Territórios Populares.

[4] Nunes N.R.d.A., Veillette A.-M. (2022). Mulheres de Favelas e O (Outro) Feminismo Popular. Rev. Estud. Fem..

[5] Fonseca D.P.R.d., Lima T.M.d.O. (2012). Outras Mulheres: Mulheres Negras Brasileiras Ao Final da Primeira Década do Século XXI.

[6] Del Priore M. (2009). Ao Sul do Corpo: Condição Feminina, Maternidades e Mentalidades No Brasil Colônia.

[7] Valadares L. (2005). A Invenção da Favela: Do Mito de Origem da Favela.

[8] Lannes-Fernandes F. (2012). Os Jovens da Favela. Reflexões Sobre Controle e Contenção Sócio-Espacial Dos Párias Urbanos No Rio de Janeiro. Convergência. Rev. Cienc. Soc..

[9] Telles V.d.S. (2006). Favela, Favelas: Interrogando Mitos, Dogmas e Representações. Rev. Bras. Ciênc. Sociais.

[10] Buss P.M., Pellegrini A.F. (2007). A Saúde e seus Determinantes Sociais. Physis Rev. Saúde Coletiva.

[11] Mattioni F.C., Silveira R.d.P., Souza C.D.d., Rocha C.M.F. (2022). Práticas de promoção da saúde como resistência e contraconduta à governamentalidade neoliberal. Ciênc. Saúde Coletiva.

[12] World Health Organization (2008). International Commission on Social Determinants of Health.

[13] World Health Organization (2011). Social Determinants of Health: Outcome of the World Conference on Social Determinants of Health (Rio de Janeiro, Brazil, October 2011).

[14] Hill-Briggs F., Adler N.E., Berkowitz S.A., Chin M.H., Gary-Webb T.L., Navas-Acien A., Thornton P.L., Haire-Joshu D. (2020). Social Determinants of Health and Diabetes: A Scientific Review. Diabetes Care.

[15] McGreevy M., Harris P., Delaney-Crowe T., Fisher M., Sainsbury P., Riley E., Baum F. (2020). How Well do Australian Government Urban Planning Policies Respond to the Social Determinants of Health and Health Equity?. Land Use Policy.

[16] Spiegel J.M., Breilh J., Yassi A. (2015). Why Language Matters: Insights and Challenges in Applying a Social Determination of Health Approach in a North-South Collaborative Research Program. Glob. Health.

[17] Breilh J. (2021). La Categoría Determinación Social Como Herramienta Emancipadora: Los Pecados de La “Experticia”, a Propósito Del Sesgo Epistemológico de Minayo. Cad. Saúde Pública.

[18] Lee K., Freudenberg N. (2022). Public Health Roles in Addressing Commercial Determinants of Health. Annu. Rev. Public Health.

[19] Dall’Alba R., Rocha D.G. (2021). Brazil’s Response to COVID-19: Commercial Determinants of Health and Regional Inequities Matter. Lancet Glob. Health.

[20] Anaf J., Baum F., Fisher M., Friel S. (2019). Civil Society Action against Transnational Corporations: Implications for Health Promotion. Health Promot. Int..

[21] Wallerstein N., Duran B., Oetzel J.G., Minkler M. (2017). Community-Based Participatory Research for Health: Advancing Social and Health Equity.

[22] Velasco H., Díaz de Rada A. (1997). La lógica de la Investigación Etnográfica.

[23] Biernacki P., Waldorf D. (1981). Snowball Sampling: Problems and Techniques of Chain Referral Sampling. Sociol. Methods Res..

[24] Albuquerque E.M. (2009). Avaliação da Técnica de Amostragem “Respondent-Driven Sampling” na Estimação de Prevalências de Doenças Transmissíveis Em Populações Organizadas em Redes Complexas. Master’s Thesis.

[25] Souza L.K.d. (2019). Pesquisa com Análise Qualitativa de Dados: Conhecendo a Análise Temática. Arq. Bras. Psicol..

[26] Braun V., Clarke V. (2006). Using Thematic Analysis in Psychology. Qual. Res. Psychol..

[27] Haesbaert R. (2004). O Mito da Desterritorialização: Do “fim dos Territórios” à Multi-Territorialidade.

[28] Lefebvre H. (1980). A Vida Cotidiana no Mundo Modern.

[29] Biroli F. (2015). Responsabilidades, Cuidado e Democracia. Rev. Bras. Ciência Política.

[30] Miguel L.F., Biroli F. (2014). Feminismo e Política: Uma Introdução.

[31] Souto S. (2021). É Tempo de Aquilombar: Da Tecnologia Ancestral à Produção Cultural Contemporânea. Políticas Cult. Rev..

[32] Nascimento M.B. (2006). O Conceito de Quilombo e a Resistência Cultural Negra. Eu Sou Atlântica: Sobre a Trajetória de Vida de Beatriz Nascimento.

[33] Brasil Ministério da Saúde, Secretaria de Vigilância em Saúde, Secretaria de Atenção à Saúde (2006). Política Nacional de Promoção da Saúde (PNPS) Portaria MS/GM No 687, de 30 de Março de 2006.

[34] Casemiro J.P., Fonseca A.B.C., Secco F.V.M. (2014). Promover Saúde na Escola: Reflexões a Partir de Uma Revisão sobre Saúde escolar na América Latina. Ciência Saúde Coletiva.

[35] Goes E.F., Ramos D.d.O., Ferreira A.J.F. (2020). Desigualdades raciais em saúde e a pandemia da COVID-19. Trab. Educ. Saúde.

[36] IBGE (2021). Coordenação de População e Indicadores Sociais. Síntese de Indicadores Sociais: Uma Análise Das Condições de Vida da População Brasileira: 2021.

[37] Crenshaw K. (2002). Documento Para O Encontro de Especialistas Em Aspectos da Discriminação Racial Relativos Ao Gênero. Rev. Estud. Fem..

[38] Kilomba G. (2019). Memórias da Plantação: Episódios de Racismo Cotidiano.

[39] Bandini M., Camargo A.C.G. (2015). Competências Requeridas Para Os Promotores de Saúde. Revista Proteção.

[40] Hooks B., Maringolo C.B. (2019). Erguer a Voz: Pensar Como Feminista, Pensar Como Negra.

[41] Mattioni F.C., Rollo R.M., Silveira R.d.P., Dresch L.C., Rocha C.M.F. (2022). Métodos Participativos: Um Caminho Para a Promoção da Saúde. Métodos Participativos em Promoção da Saúde.

